# Stability of microRNAs in serum and plasma reveal promise as a circulating biomarker

**DOI:** 10.1016/j.ncrna.2025.08.001

**Published:** 2025-08-08

**Authors:** Erryk S. Katayama, Jonathan J. Hue, Alexander W. Loftus, Semmer A. Ali, Hallie J. Graor, Luke D. Rothermel, Eric Londin, Mehrdad Zarei, Jordan M. Winter

**Affiliations:** a Case Comprehensive Cancer Center, Case Western Reserve University, Cleveland, OH, USA; b Department of Biochemistry, Case Western Reserve University, Cleveland, OH, USA; c The Ohio State University College of Medicine, Columbus, OH, USA; d Department of Surgery, Division of Surgical Oncology, University Hospitals Cleveland Medical Center, Cleveland, OH, USA; e Department of Pathology, Anatomy and Cell Biology, Thomas Jefferson University, Philadelphia, PA, USA

**Keywords:** Disease diagnosis, microRNA, Blood, Biomarker, Early detection

## Abstract

**Purpose:**

To verify the stability and reliability of circulating microRNA (miRNA) profiles in plasma and serum under different processing and storage conditions to inform future applications to circulating biomarker analyses.

**Background:**

The development of blood-based methods for early disease detection has become increasingly desirable across various medical fields. RNA profiles have been investigated but have been a challenge due to rapid degradation of the analyte by ubiquitous RNases. miRNAs are short, non-coding regulatory RNAs that are believed to be more stable under certain conditions, large in number, and specific to cell type and disease state. Thus, circulating miRNA profiles hold significant promise as diagnostic biomarkers for a range of conditions, including cancer, autoimmune, liver, neurological, metabolic, and cardiovascular diseases. However, to realize their full potential in clinical applications, it is crucial to thoroughly characterize the stability of miRNAs under various blood collection, processing, and storage conditions prior to their investigation and large-scale application in disease-specific biomarker discovery studies.

**Methods:**

Plasma or serum were extracted from whole blood of healthy volunteers. Samples were stored at different temperatures (4 °C or 25 °C, room temperature) for varying periods (0–24 h) to mimic possible delays in processing encountered in routine clinical settings. miRNA was extracted and profiles were assessed with RT-qPCR or small RNA-sequencing techniques.

**Results:**

Mean Cq values of specific miRNAs, such as miR-15b, miR-16, miR-21, miR-24, and miR-223, remained consistent between 0 and 24 h when serum and plasma were stored on ice. Minimal changes were observed in mean Cq values over 24 h when serum was left at room temperature as well. Similar trends were observed when miRNAs from plasma were analyzed. Small-RNA sequencing detected approximately ∼650 different miRNA signals in plasma, with over 99 % of the miRNA profile unchanged even when blood draw tubes were left at room temperature for 6 h prior to processing.

**Conclusions:**

These data demonstrate remarkable stability of miRNAs over time, which should withstand variability in handling and processing that can occur with routine clinical lab draws. Considering the large diversity of miRNAs, this analyte class should be thoroughly investigated as a non-invasive biomarker of diverse disease states.

## Graphical abstract

**Figure:**
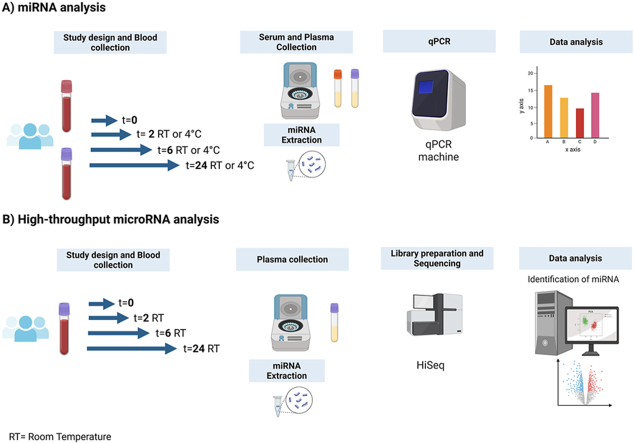

## 1 Introduction

Early disease detection is a prioritized research goal, with the hope that treatment inception earlier in the disease course can improve outcomes and reduce healthcare expenditures. This principle is relevant across a wide range of conditions, including cancer, cardiovascular disease, autoimmune disorders, liver disease, and neurologic pathology. Selected cancers offer successful examples of effective disease screening, and include mammography for breast cancer, colonoscopy for colon cancer, and the Pap Test for cervical cancer. Examples of screening in non-cancer diseases include fasting blood glucose testing for diabetes, blood pressure monitoring for hypertension, cholesterol testing for cardiovascular disease, DEXA scans for osteoporosis, and questionnaires for depression screening [[1], [2], [3], [4], [5], [6], [7], [8], [9]]. Some screening tests are used in specific high risk populations, including coronary artery calcium scoring, PSA for prostate cancer, CT for patients at increased risk for lung cancer, and HbA1c levels for type 2 diabetes [[10], [11], [12], [13]].

Some approaches under investigation screen for symptoms, such as unintentional weight loss, prodromal depression, or pancreatogenic diabetes for pancreatic and other cancers [14,15]; persistent cough or hemoptysis for lung cancer; unexplained fatigue for liver disease; or headaches for neurological disorders [[16], [17], [18]]. While helpful in triggering diagnostic evaluations, symptom based-screening lacks the accuracy to uncover diagnoses without complementary investigations. In recent years, investigators have tested blood-based biomarkers for this purpose. A variety of circulating analytes have been interrogated towards this end, including genomic DNA, circulating tumor cells, and protein antigens [19]. Methylated DNA signatures in the plasma for example have recently emerged as a clinically available multi-cancer early detection test [20,21]. The utilization of circulating RNAs for this purpose is perhaps less well described [[22], [23], [24]] and is fraught with perceived analyte stability challenges.

mRNA transcripts are typically more than 2 kb long, and therefore susceptible to rapid degradation by RNases [[24], [25], [26], [27]]. However, microRNAs (miRNA) are short oligonucleotide sequences which may be more resistant to degradation, and therefore are an RNA subtype with perhaps greater promise as a potential biomarker of human disease [28]. miRNAs are around 21 to 25 nucleotides in length, and do not encode proteins. Instead, they have a regulatory role, typically at the post-transcriptional level of gene processing. These analytes are detectable in virtually all biofluids and are often packaged within exosomes or complexed with proteins for added stability [23,27,[29], [30], [31], [32]]. Additionally, recent studies reveal a very large variety of small RNAs, which offers the possibility that distinct signatures or patterns exist which may reveal disease-specific information [33]. Indeed, it is likely that these small RNAs even contribute to the generation of specific disease states (including cancer) or factor into the host response to disease, and so investigations could have the added benefit of uncovering biologic insights into pathogenesis.

While miRNAs are considered more stable than longer RNAs, quality control validation steps are still necessary to establish proof of concept for miRNA utility as a potential circulating biomarker, prior to performing large-scale and expensive biomarker discovery studies [34,35]. Concerns with RNA stability require rigorous assessment of even small RNAs under practical, real-world, blood drawing conditions [27,29,36]. Recent work by Sandau et al. in a larger cohort reinforces the relevance of this issue, demonstrating that pre-analytical handling variables significantly affect small RNA profiles, including miRNAs, despite the use of standardized protocols [37]. Prior studies have already tested the stability of a small handful of specific circulating miRNAs in the blood revealing favorable results, yet the stability of the larger untargeted miRNAome at scale has not been tested to our knowledge. Herein, we characterize miRNA stability, including at the microRNAome level, in both serum and plasma as a foundational step for future biomarker discovery studies and the possible application of miRNAs for clinical biomarker usage.

## 2 Methods

### 2.1 Institutional assurances

Blood samples were drawn from volunteer participants with informed written consent under a protocol approved by the Institutional Review Board (IRB) at Case Western Reserve University (IRB Number: STUDY20200198).

### 2.2 Sample selection and inclusion criteria

The five samples were obtained from healthy volunteers recruited sequentially under the approved IRB protocol STUDY20200198. No samples were excluded from the study. All donors who provided informed consent during the recruitment window and supplied sufficient blood volume for both serum and plasma analyses were included. The five individuals (2 females, 3 males) ranged in age from 24 to 51 years. All were non-smokers, not taking anticoagulants, and had no known acute or chronic medical conditions at the time of blood collection. Exclusion criteria included recent infection, active inflammation, or medication use known to affect circulating RNA profiles.

### 2.3 RNA extraction

*miRNA Isolation:* miRNA was extracted from plasma and serum using Qiagen miRNeasy Serum/Plasma Kit (Qiagen, 217184) according to manufacturer's protocol, with minor adjustments to the final elution volume (28 vs 14 μL of water) and centrifugation time (2 vs 1 min).

*Total RNA Isolation:* Control RNA from MIAPaCa-2 cells was extracted using PureLink RNA isolation kit (Life Technologies, 12183025) according to the manufacturer's protocol optimized for use with TRIzol® reagent [38].

### 2.4 Reverse transcription and quantitative real-time polymerase chain reaction

cDNA was synthesized using the Applied Biosystems High-Capacity RNA-to-cDNA kit (Thermo Fisher, 01127021). RT-qPCR for mRNA was performed using primers for human 18S (Thermo Fisher, Hs99999901_s1) and β-Actin (Thermo Fisher, Hs01060665_g1). RT-qPCR for miRNA was performed using TaqMan MicroRNA Assay (Thermo Fisher, 4427975) with primers for hsa-miR-15b (Assay ID 000390), hsa-miR-16 (Assay ID 000391), hsa-miR-21 (Assay ID 000397), hsa-miR-24 (Assay ID 000402), and hsa-miR-223 (Assay ID 002295). miRNA primer target sequences are tabulated in Supplementary Table S1. Cell-based 18S was used as a positive control for all PCR reactions. All PCR reactions were performed in triplicate using iTaq Universal Probes Supermix (Bio-Rad, 1725131). RT-qPCR acquisition was performed using a Bio-Rad C1000 Touch™ Thermal Cycler and CFX96™ Real-Time System.

### 2.5 Blood sample preparation for targeted miRNA analysis via RT-qPCR

Whole blood was collected in a single 10 mL K2EDTA tube (plasma, purple tops) or a single 10 mL clotting tube (serum, red tops) per sample. Serum samples were left to clot at room temperature for 30 min before centrifugation. Both plasma and serum samples were centrifuged at 1200×*g* for 10 min at room temperature. The top layer of sample was carefully collected, transferred to a new centrifuge tube, and centrifuged at 1500×*g* for an additional 5 min at room temperature. Aliquots of plasma and serum samples (0.5 mL) were pipetted into 1.5 mL microcentrifuge tubes. For indicated experiments, plasma and serum samples for miRNA analysis were left for 0–24 h on ice or at room temperature to evaluate the effect of processing time on RNA stability. All samples were subsequently stored at −80 °C for future experiments probing for specific, individual miRNAs.

### 2.6 Blood sample preparation for high throughput small-RNA sequencing

#### 2.6.1 Sample collection

Peripheral blood was collected from two healthy volunteers in three to four 10 mL K2EDTA tubes per replicates, within each time point, yielding a total plasma volume of 15–20 mL per time point, across three separate replicates. Plasma was isolated immediately after centrifugation and allowed to sit for 0, 6, and 24 h at room temperature prior to sequencing. Additionally, a separate set of samples was collected from a healthy volunteer to further assess the impact of processing delays on plasma isolation. For this set, whole blood was left at room temperature for 0, 2, and 6 h before isolating plasma.

After the designated time points, all blood samples were centrifuged at 1200×*g* for 10 min at room temperature. Plasma was carefully isolated, and hemolysis was evaluated by visual inspection, with a pink discoloration indicating the presence of free hemoglobin and suggesting a risk of hemolysis.

#### 2.6.2 Small RNA library preparation, multiplexing and cluster generation

RNA samples were quantified using Qubit 2.0 Fluorometer (Life Technologies) and RNA integrity was checked with 4200 TapeStation (Agilent Technologies). RNA library preparations and sequencing reactions were conducted at GENEWIZ, LLC. (South Plainfield, NJ, USA). Small RNA sequencing libraries were prepared using Illumina TruSeq Small RNA library Prep Kit (Illumina). In brief, Illumina 3′ and 5′ adapters were added to RNA molecules with a 5′-phosphate and a 3′-hydroxyl group sequentially. A reverse transcription reaction was used to create single stranded cDNA. cDNA was then PCR amplified using a common primer and a primer containing the index sequence. Amplified cDNA constructs were purified by polyacrylamide gel electrophoresis, and the correct band (∼145–160 bp) was excised from the gel and eluted with water. The eluted cDNA was concentrated by ethanol precipitation. The sequencing library was validated on the Agilent TapeStation (Agilent Technologies) and quantified using Qubit 2.0 Fluorometer (Invitrogen) as well as by quantitative PCR (KAPA Biosystems).

#### 2.6.3 Sequencing

The sequencing libraries were multiplexed and clustered onto a flowcell. After clustering, the flow cell was loaded onto the Illumina HiSeq instrument according to manufacturer's instructions. The samples were sequenced using a 2 × 150 bp Paired End (PE) configuration. Image analysis and base calling were conducted by the HiSeq Control Software (HCS). Raw sequence data (.bcl files) generated from Illumina HiSeq was converted into FASTQ files and de-multiplexed using Illumina bcl2fastq 2.17 software. One mismatch was allowed for index sequence identification.

### 2.7 Statistical analysis

The data are expressed as mean ± SEM (standard error of the mean) of at least three independent experiments unless indicated. mRNA levels were calculated using the ΔΔCt method, as previously described [39]. miRNA Cq values were obtained by RT-qPCR and comparison between groups were determined using One-way ANOVA. GraphPad Prism 10.0.02 Software was used for statistical analysis. miRNA-sequencing data were assessed using FastQC [40]. Reads were further aligned to Homo sapiens genome build hg19 using miRDeep2 for mature miRNA expression analysis [41]. miRDeep2 alignments were normalized via Relative Log Expression (RLE) using DESeq2 R [42]. Principle component analysis (PCA) was used to examine covariance and data structure in miRNA profiles of samples. StataSE v16.1 (Statacorp LLC, College Station, TX) was used for statistical analysis. For final analyses, a p-value was considered statistically significant when <0.05.

## 3 Results

### 3.1 Calibration of miRNA and degradation of mRNA over time

**Fig. 1:**
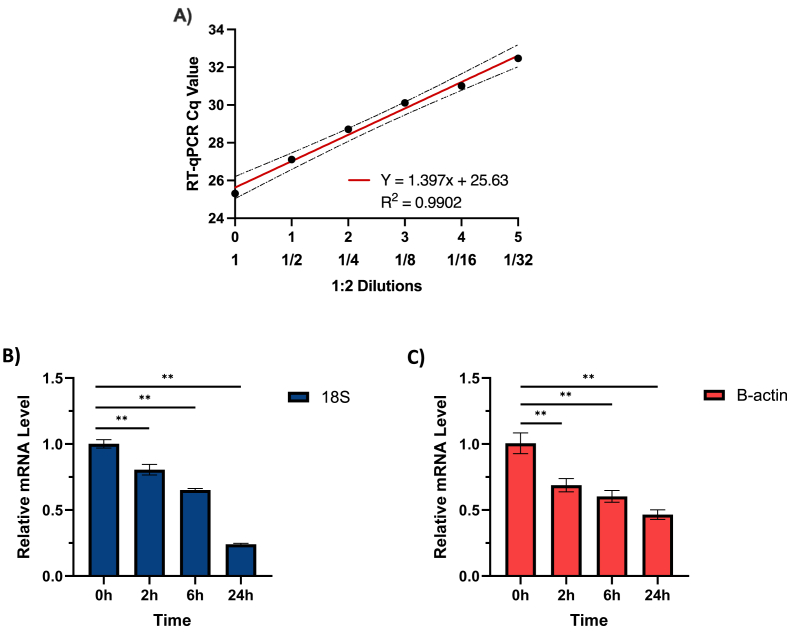
**Calibration of cDNA and degradation of mRNA over time.A)** Serial dilution of miR-16 cDNA to demonstrate that Cq correlates with relative cDNA concentration. **B–C)** Degradation of house-keeping mRNAs 18s (B) and β-Actin (C) in plasma at room temperature at indicated time points. Comparisons between groups were determined using an unpaired, two-tailed Student's *t*-test (∗p < 0.05; ∗∗p < 0.01).

Serial dilutions of miR-16 cDNA were performed to calibrate quantitation using qPCR. There was a linear increase in Cq value as the quantity of cDNA decreased. A slope of 1.397 indicated a roughly 40 % reduction in miR-16 quantity with each 50 % dilution, and the y-intercept of 25.6 indicates the Cq value corresponding to the baseline level of noise in the assay (Fig. 1A). We next examined coding mRNA degradation over time to serve as a reference point for eventual miRNA degradation experiments. As expected, there was a persistent decrease in relative β-Actin (1.2 kb) and 18S (1.9 kb) mRNA levels over time, as representative coding mRNAs. The exact decrements in mRNA levels at 2, 6, and 24 h compared to baseline (0 h), were 0.2 and 0.3; 0.35 and 0.4; and 0.77 and 0.57 for 18S and β-actin, respectively (Fig. 1B).

### 3.2 Targeted miRNA analyses of blood samples stored on ice

**Fig. 2:**
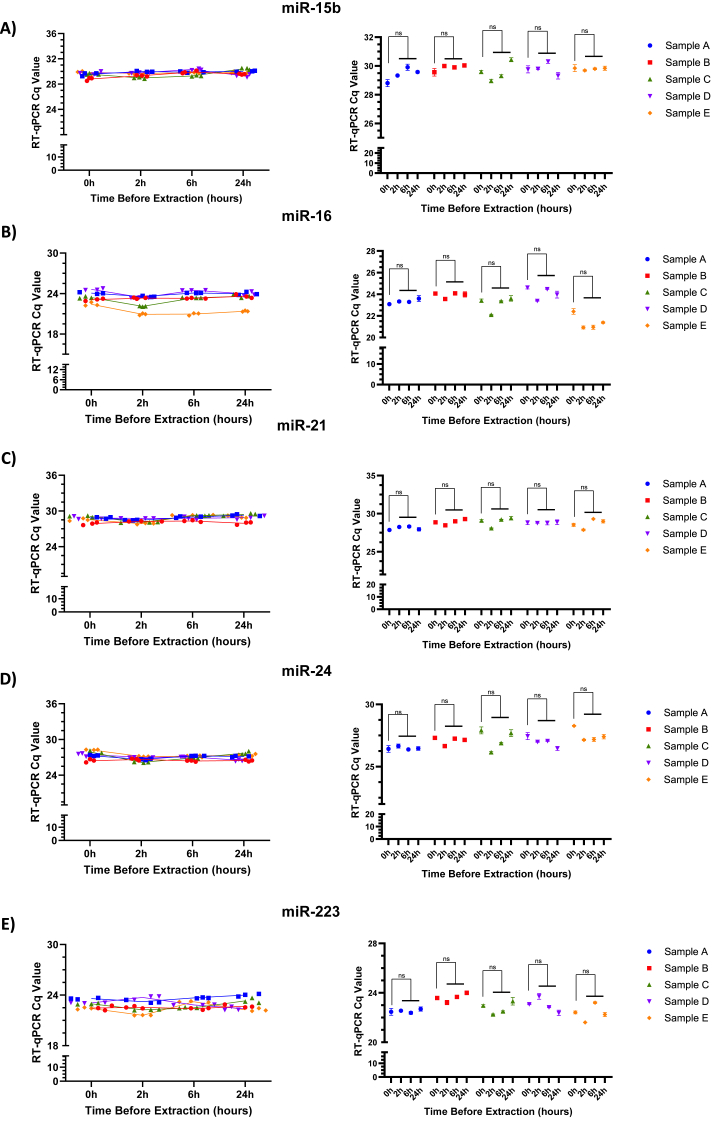
**Stability of miRNAs in serum samples stored on ice at different time points.A–E)** Raw Cq values of five individual miRNAs (miR-15b, miR-16, miR-21, miR-24, and miR-223), obtained via RT-qPCR of serum samples stored on ice at 0, 2, 6, and 24 h. Data are representative of five individuals tested in triplicate at each time point. Each sample (A–D) represents a separate individual, and the mean of triplicate values are presented in the right side of the figure. Comparisons between groups were determined using an unpaired, two-tailed Student's *t*-test (ns; nonsignificant).

Selected miRNAs were analyzed for stability as proof of concept, and were chosen based on their known expression across multiple cancer models, as well as cardiovascular and inflammatory diseases [[43], [44], [45], [46], [47], [48]]. Target miRNAs were prepared from blood draws of healthy volunteers and both plasma and serum were allowed to sit on ice for the indicated times prior to miRNA extraction, which tested potential time variability in the sample preparation process. A parallel experiment conducted at room temperature is described below. Mean Cq values for specific miRNAs from serum samples stored on ice remained consistent at time points over the course of 24 h, including the beginning and end of the experiment (Fig. 2A–E). Each sample (A–E) represents all of the time points from a given individual. As shown, miR-15b, miR-16, miR-21, miR-24, and miR-223 were stable in serum over all of the tested time intervals in each individual or sample set. While there were variations in Cq values between subjects, the overall trends in miRNA stability over time were similar across all cases.

Mean Cq values for plasma stored on ice at the same time points exhibited a similar trend of stability. Tested miRNAs demonstrated statistical significance in one of the samples out of five individuals, although most samples’ Cq values over the 24 h period were consistent (Supplementary Fig. S1A–E).

### 3.3 Targeted miRNA analyses of blood samples stored at room temperature

**Fig. 3:**
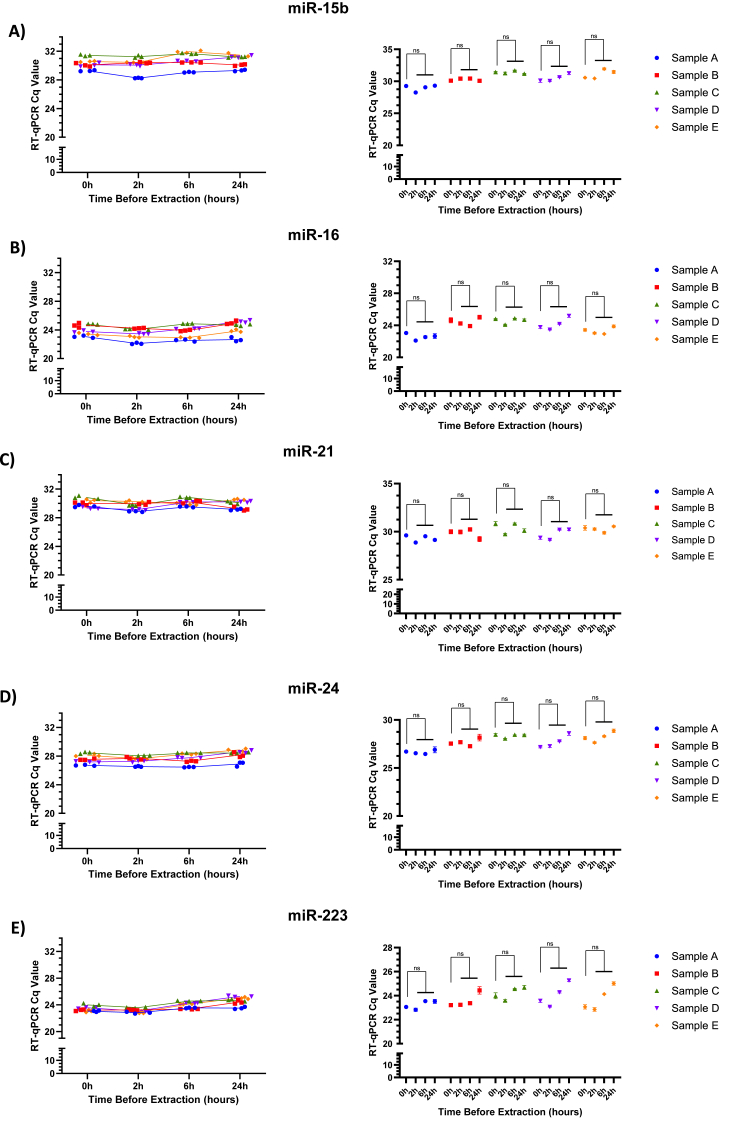
**Stability of miRNAs in serum samples stored at room temperature at different time points. A–E)** Raw Cq values of five individual miRNAs (miR-15b, miR-16, miR-21, miR-24, and miR-223), obtained via RT-qPCR of serum samples stored at room temperature for 0, 2, 6, and 24 h. Data are representative of five individuals tested in triplicate at each time point. Each sample (A–D) represents a separate individual, and the mean of triplicate values are presented in the right side of the figure.

We then analyzed the stability of selected miRNAs in serum and plasma when stored at room temperature. The mean Cq values of the tested miRNAs in serum, including miR-15b, miR-16, miR-21, miR-24, and miR-223 (Fig. 3A–E), remained stable over the course of 24 h. Similarly, in plasma samples, the miRNAs miR-15b, miR-16, miR-21, miR-24, and miR-223 demonstrated remarkable stability when incubated at room temperature for 2, 6, and 24 h (Supplementary Fig. S2A–E). Thus, minimal variability in Cq values across time points indicates that these specific miRNAs have the potential to serve as robust circulating markers if biologically indicated, demonstrating stability in both serum and plasma during routine handling and even delayed processing.

### 3.4 miRNAome sequencing analysis

**Fig. 4:**
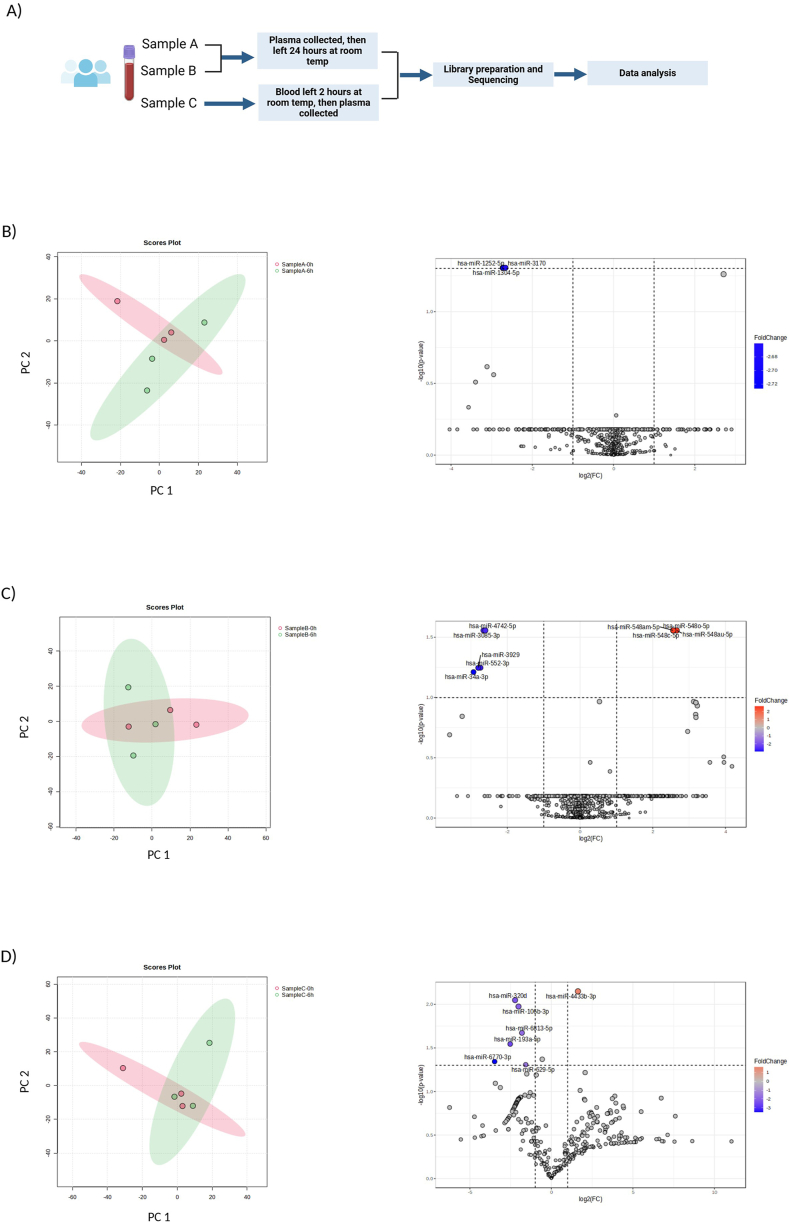
**Small RNA-sequencing of miRNA present in plasma samples from three individuals.A)** Workflow for sample preparation and analysis. **B &C)** After blood was collected and plasma was extracted, plasma samples from two individuals were set at room temperature for either 0 or 6 h and miRNAs from the plasma were analyzed. **B)** Principal component analysis (left) and volcano plot (right) comparison of sample A at 0 h to sample A at 6 h. **C)** Comparison of sample B at 0 h to sample B at 6 h. **D)** Blood was drawn and set at room temperature for either 0 or 6 h, and then plasma was extracted and miRNAs from the plasma analyzed. Comparison of sample C at 0 h to sample C at 6 h. Differential expression analysis of miRNA transcriptome is summarized as a volcano plot with an FDR rate of <0.05 and fold change of ≥2. Down-regulated miRNAs are depicted in blue and up-regulated miRNAs are depicted in red.

We performed small-RNA sequencing of plasma samples from two individuals and were able to detect ∼650 different miRNAs in each sample out of a potential probe set of 2500. miRNAs below the level of detection were likely not present or were present at levels below the level of detection. In this experiment, blood was drawn from each individual and plasma was extracted immediately, and then allowed to sit at room temperature for either 0, 6 or 24 h before the miRNA was extracted and processed (Fig. 4A and Supplemental Fig. S3A). Experiments were performed in triplicate for each individual.

Profiles of these miRNAs changed only minimally after 6 h at room temperature, with only three diminished miRNAs in sample A, accounting for less than 1 % of total miRNA detected (Fig. 4B). No miRNAs were statistically increased. In the sample set from the second individual, we observed just two decreased miRNAs, accounting for 0.5 % of total miRNAs, and increased levels in four miRNAs, accounted for less than 1 % of total miRNAs (Fig. 4C). The fact that some miRNAs increased in levels during the study interval suggests that these rare changes reflected small degrees of noise or imprecision in the analysis and were not a function of degradation or accumulation over time. Overall, we observed stability in 99.2 % of detectable miRNAs over 6 h when samples were allowed to sit on room temperature before processing. We repeated the comparison after 24 h at room temperature. Plasma from individual A showed a decrease in five miRNAs, accounting for 1 % of the total miRNAs and an increase in eight miRNAs accounting for less than 2 % of the total miRNAs (Supplemental Figure S3B). Plasma from individual B showed five miRNAs with increased levels, accounting for 1 %, and none with diminished levels (Supplemental Figure S3C). Once again, the fact that comparable numbers of miRNA reductions and increases were observed suggests random variation and minimal noise in the detection process, as opposed to detection of degradation over time. At least 99 % of the miRNAs were detected to be stable at room temperature over 24 h.

We performed the experiment separately in one individual, changing the variable of processing time to occur at the level of the blood draw (earlier in the processing workflow), instead of after plasma preparation. The tube of blood in this case was set at room temperature for either 0, 2 or 6 h, and then subsequently plasma was extracted and miRNAs from the plasma were analyzed (Fig. 4A and Supplemental Fig. S3A). This protocol was experimentally identical to the studies of specific miRNAs detailed above with respect to the processing delay variable. Minimal changes in total detectable miRNAs were observed after 2 h, with only one miRNA diminished and one increased over time (Supplementary Fig. S3D). After 6 h, the profiles showed only slightly more variation, with six miRNAs decreasing in levels, accounting for less than 1 % of the total miRNAs, and increased levels in one miRNA (Fig. 4D). The miRNAs with statistically significant differences showed no overlap between individuals, nor did they reflect a distinct pattern of miRNA change between different time points of the same individuals, suggesting again that the observed changes were merely a reflection of a small amount of background noise. The detected miRNAs were then ranked by their baseline expression levels in Counts per Million (CPM) (Supplemental Fig. S4A and B) to determine if changes over time were a function of miRNA quantity in the sample. One might suspect that more abundant miRNAs might be prone to fluctuations in levels across time points. Here, we see that miRNAs at low and high expression levels were fairly well distributed in the dataset. Additionally, we analyzed those rare miRNAs that were detected at decreased or increased levels at later time points. Notably, most of the noise was observed at low CPM values, and all detected changes fell within this category, suggesting that these variations could potentially be attributed to technical noise rather than biological significance. The data revealed that stability was observed both in high-expression and low-expression miRNAs. Thus, this analysis further indicated a relatively high degree of precision in detection capability, with minimal noise.

## 4 Discussion

The concept of liquid biopsies for disease detection dates back to the 1930s [[49], [50], [51]], with a wide range of blood components investigated as potential biomarkers over the years, including miRNAs, proteins, small-molecule metabolites, DNA, circulating RNA, and circulating tumor cells. Circulating protein antigen expression has been the gold standard test in this space, especially for cancer, because these markers are readily detectable through ELISA assays. Notable examples include prostate-specific antigen (PSA) for prostate cancer, carbohydrate antigen (CA) 19-9 for pancreatic cancer, CA125 for ovarian cancer, and carcinoembryonic antigen (CEA) for colorectal and other cancers [2,3,[52], [53], [54], [55]]. With the exception of PSA, these circulating biomarkers are utilized to monitor disease burden, and are not effective as cancer screening tests. Thus, most diseases, benign and malignant, lack validated protein biomarkers even as indicators of advanced disease, and there remain a paucity of screening tests for early disease detection. The pursuit of novel and effective biomarkers remains a crucial one to inform biology, enable early detection, and guide treatment [56,57].

Advances in spectrometry methodologies have opened the door to plasma and serum metabolite profile-based screening. While studies have begun to investigate unique metabolic profiles for various diseases, challenges remain due to confounding environmental factors (i.e., diet, lifestyle) and the high cost of equipment [52,58,59]. Circulating cells and secreted nucleic acids represent novel advancements moving into clinical development. For instance, current trials are validating the efficacy of tests that detect methylation patterns of cell-free DNA indicative of certain disease states (e.g., cancer) and some commercial tests are already available [60,61].

miRNA-sequencing in tissues has been utilized in numerous studies to aid in the classification of various disease subtypes, prognosis, and response to certain therapies. Its use as a broad-based screening test has only been superficially explored [62,63]. miRNAs are believed to have a relatively longer half-life than other analyte categories under investigation, ranging from as short as 4 h to as long as 24 h [32,[64], [65], [66], [67]]. Herein, we validate and expand upon past reports and demonstrate remarkable stability of five separate miRNAs in plasma and serum using different processing time points and conditions. Our intention was to simulate processing delays that could occur in ‘real world’ laboratory scenarios to determine if miRNA stability under those conditions were sufficient as a potential early detection marker to warrant larger scale biomarker discovery studies. Indeed, miRNA profiles remained extremely stable, even when samples were left at room temperature for as long as 24 h.

To our knowledge, the present studies are among the first to characterize stability of miRNA profiles beyond targeted miRNAs, and instead at the miRNA-omic level [68]. The data suggest that a subject's entire circulating miRNA signature is stable over time relevant to actual clinical processing workflows, making this analyte class an ideal candidate for further investigation. The added step of observing stability at the microRNAome level, rather than specific miRNAs, enables a broader panel of miRNAs to be assessed in a high throughput way, and more importantly, identify signatures of microRNAs that may be informative as markers of disease. Many of the ∼650 detected miRNAs were present at moderate-to-high abundance and likely share features such as packaging in exosomes, binding to Argonaute proteins, or association with lipoprotein particles, which confer stability in circulation. The few miRNAs showing variability tended to have low baseline expression (low CPM), suggesting that abundant miRNAs are inherently more stable due to biological protection and reduced technical noise, supporting their potential as reliable biomarkers.

We acknowledge the detection of occasional statistically significant differences in miRNA quantity over time in these studies. These were rare observations, both in the targeted miRNA and microRNAome studies. We hypothesize that these variations could be due to miRNA degradation (i.e., instability) or merely represent an artifact of the extraction process itself. Previous literature suggests the latter scenario is a factor [27,[69], [70], [71]] and processing can be subject to inconsistencies in the steps or reagents [[71], [72], [73]]. In an attempt to limit these differences in extraction, a standardized kit was utilized and all experiments were performed by a single researcher. We submit that the rare differences seen more likely represent the baseline background noise of the analysis, and is roughly ±1 % of the total miRNAs examined. Additionally, we analyzed a library of 2500 miRNAS, with detection of ∼650 miRNAs above background levels across the sample set. Recent studies indicate the diversity of miRNAs is even larger, with a potential pool of miRNAs up to 5000 or more [33,41,[74], [75], [76]]. Thus, future studies interrogating this analyte (i.e., miRNA) as a potential biomarker should include methodologies that capture the full range of this diversity. Overall, miRNA stability is acceptable over processing times likely to be relevant to a clinical setting, paving the way for large scale small RNA sequencing studies across disease states to identify novel markers and signatures of disease and disease burden.

## CRediT authorship contribution statement

**Mehrdad Zarei:** Writing – review & editing, Writing – original draft, Visualization, Validation, Supervision, Resources, Project administration, Methodology, Investigation, Formal analysis, Data curation, Conceptualization.

## Availability of data and materials

Not applicable.

## Funding

Grant support for this research comes from the American Cancer Society MRSG-14-019-01-CDD, American Cancer Society 134170-MBG-19-174-01-MBG, Gateway for Cancer Research G-22-1100, NCI R37CA227865-01A1, NCI R01 CA281219, the Case Comprehensive Cancer Center GI SPORE 5P50CA150964-08, Case Comprehensive Cancer Center core grant P30CA043703, and University Hospitals research start-up package (J.M.W.). We are grateful for additional support from numerous donors to the University Hospitals Surgical Oncology Lab, including the John and Peggy Garson Family Research Fund, The Jerome A. and Joy Weinberger Family research fund, the Hieronymous Family, Robin Holmes-Novak in memory of Eugene, Brittan and Fred DiSanto, and Rosi and Saby Behar.

## Declaration of competing interest

The authors declare that they have no known competing financial interests or personal relationships that could have appeared to influence the work reported in this paper.

## Appendix A Supplementary data

Figure S1. **A-H)** RT-qPCR of plasma stored on ice at 0, 2, 6, and 24 h. The data are representative of five individuals (miR-15b, miR-16, miR-21, miR-24, and miR-223), with each sample tested in triplicate at each time point**.** Comparisons between groups were determined using an unpaired, two-tailed Student's t-test (ns; nonsignificant; ∗p < 0.05; ∗∗p < 0.01).

Figure S2. **A-H)** RT-qPCR of plasma stored at room temperature at 0, 2, 6, and 24 h. The data are representative of five individuals (miR-15b, miR-16, miR-21, miR-24, and miR-223), with each sample tested in triplicate at each time point.

Figure S3. Small RNA-sequencing of miRNA present in plasma samples from three individuals. **A)** Workflow for sample preparation and analysis. **B) & C)** Plasma samples from two individuals were set at room temperature for either 0 or 24 h, and miRNAs from the plasma were analyzed. **B)** Principal component analysis (left) and volcano plot (right) comparison of sample A at 0 h to sample A at 24 h. **C)** Comparison of sample B at 0 h to sample B at 24 h. **D)** Blood was drawn and set at room temperature for either 0 or 2 h, and then plasma was extracted and miRNAs from the plasma were analyzed. Comparison of sample C at 0 h to sample C at 2 h. Differential expression analysis of miRNA transcriptome is summarized as a volcano plot with an FDR rate of <0.05 and fold change of ≥2. Down-regulated miRNAs are depicted in blue and up-regulated miRNAs are depicted in red.

Figure S4. miRNA Ranking by Expression Levels in Counts per Millions (CPM). **A)** Out of 2500 miRNAs, the proportion of detected and undetected miRNAs. **B)** Detected miRNAs are ranked based on their baseline expression levels, from lowest to highest in Counts per Million (CPM). The expression levels are categorized into bins (0–2, 2–4, 4–8, and 8–16 CPM) to illustrate the distribution of miRNA abundance.
