# Supplementary material for: Stability of microRNAs in serum and plasma reveal promise as a circulating biomarker
Source: Noncoding RNA Res. 2025 Aug 8;15:132–41. doi: 10.1016/j.ncrna.2025.08.001 (PMC12414832; doi:10.1016/j.ncrna.2025.08.001)

Supplemental Figure-1

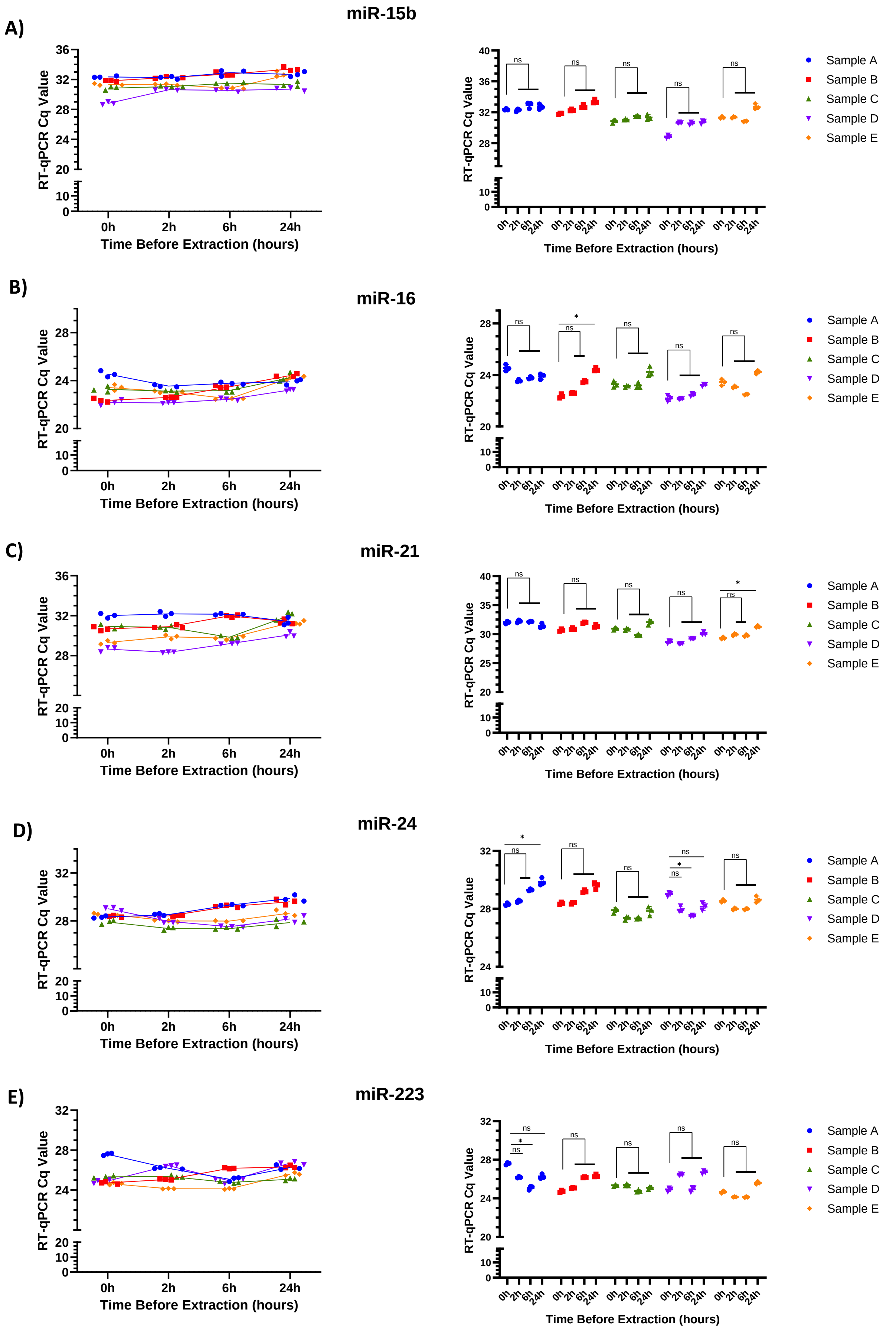

Supplemental Figure-2

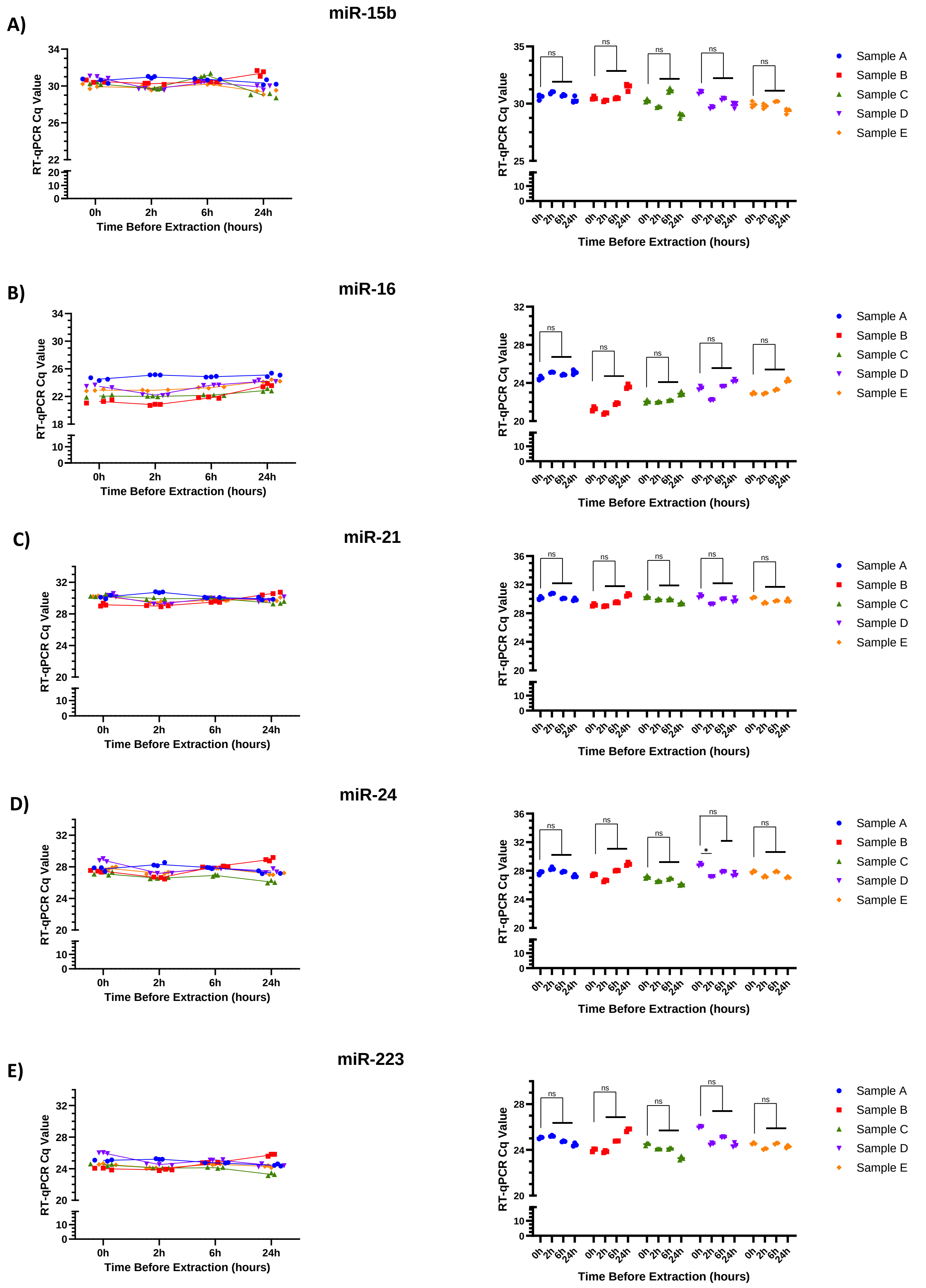

Supplemental Figure-3

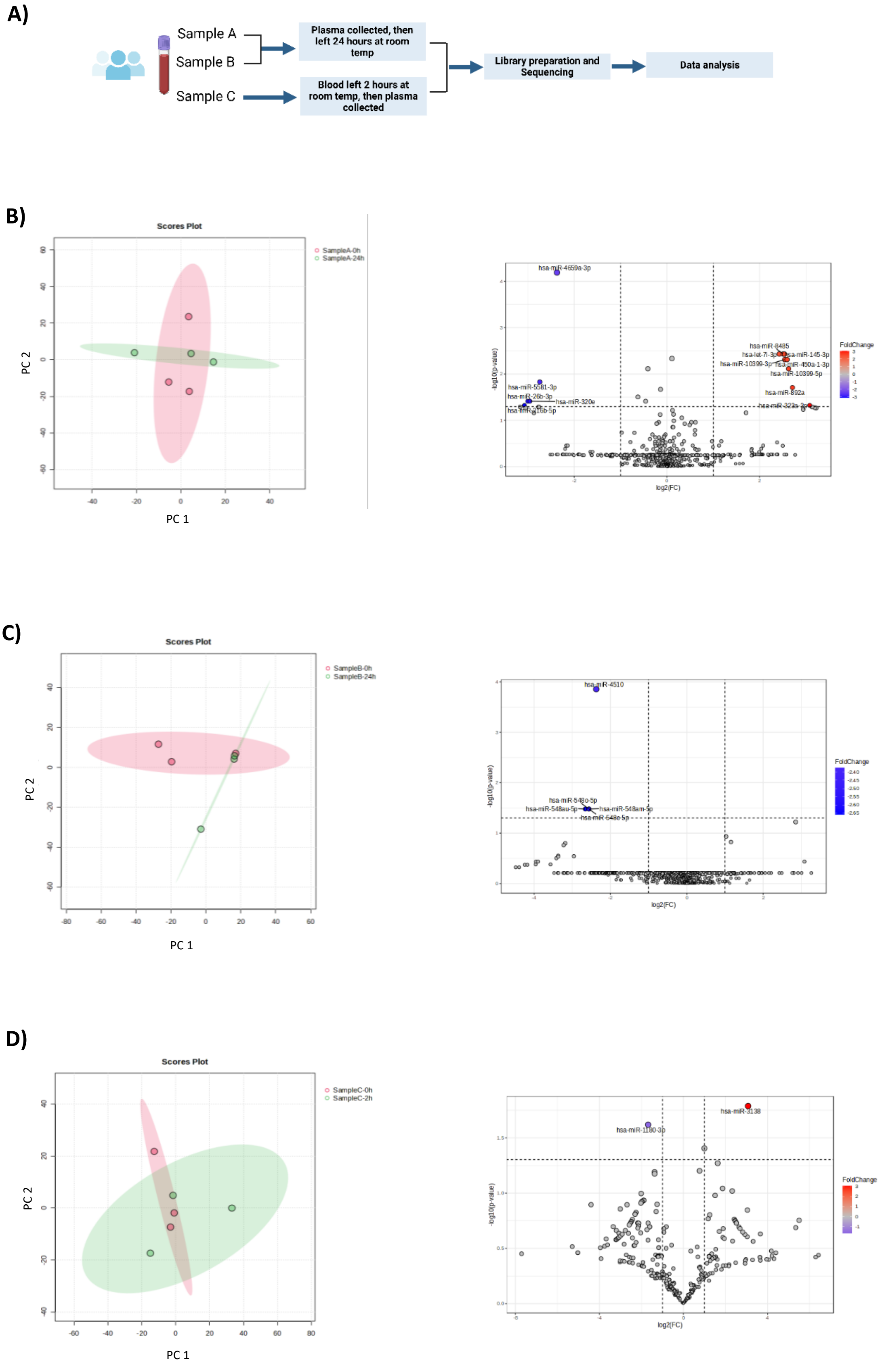

Supplemental Figure-4

A)

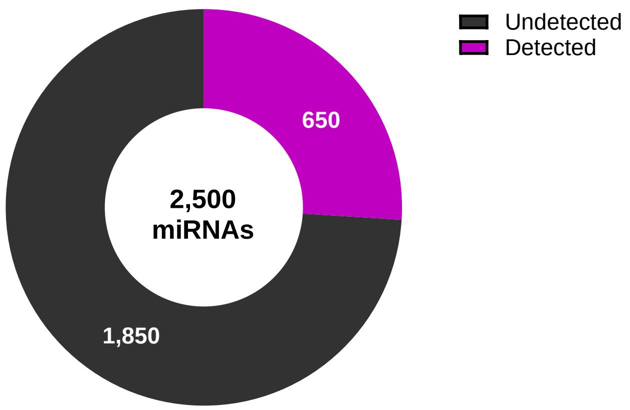

B)

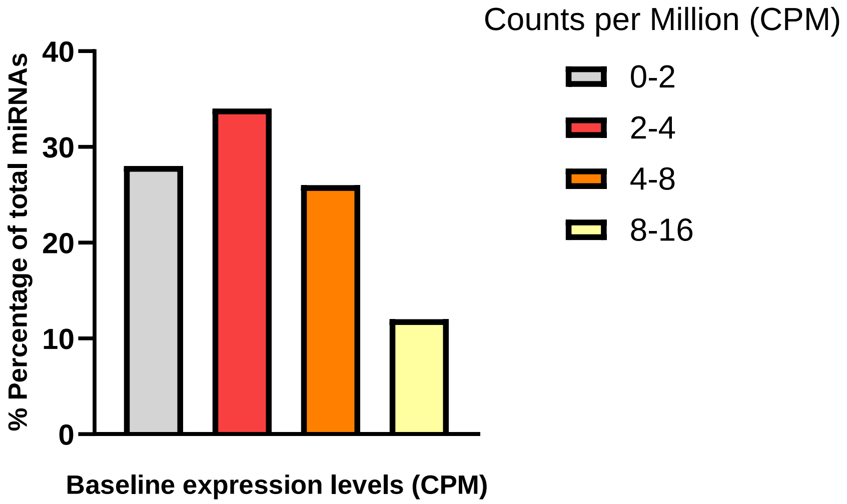

Supplement: Multimedia component 1 [file mmc1.pdf]
