# Supplementary material for: Stability of microRNAs in serum and plasma reveal promise as a circulating biomarker
Source: Noncoding RNA Res. 2025 Aug 8;15:132–41. doi: 10.1016/j.ncrna.2025.08.001 (PMC12414832; doi:10.1016/j.ncrna.2025.08.001)
Supplement: Multimedia component 2 [file mmc2.docx]

**Supplementary Data**

**Table S1**. Target sequence of each RT-qPCR miRNA primer assay from Thermo Fisher.

| **Primer** | **Catalog Number** | **Target Sequence** |
| --- | --- | --- |
| hsa-miR-15b-5p | 4427975 – Assay 000390 | UAGCAGCACAUCAUGGUUUACA |
| hsa-miR-16-5p | 4427975 – Assay 000391 | UAGCAGCACGUAAAUAUUGGCG |
| hsa-miR-21-5p | 4427975 – Assay 000397 | UAGCUUAUCAGACUGAUGUUGA |
| hsa-miR-24-3p | 4427975 – Assay 000402 | UGGCUCAGUUCAGCAGGAACAG |
| hsa-miR-223-3p | 4427975 – Assay 002295 | UGUCAGUUUGUCAAAUACCCCA |

**Figure S1. A-H)** RT-qPCR of plasma stored on ice at 0, 2, 6, and 24 hours. The data are representative of five individuals (miR-15b, miR-16, miR-21, miR-24, and miR-223), with each sample tested in triplicate at each time point**.** Comparisons between groups were determined using an unpaired, two-tailed Student’s t-test (ns; nonsignificant; * p < 0.05; ** p < 0.01).

**Figure S2. A-H)** RT-qPCR of plasma stored at room temperature at 0, 2, 6, and 24 hours. The data are representative of five individuals (miR-15b, miR-16, miR-21, miR-24, and miR-223), with each sample tested in triplicate at each time point.

**Figure S3.** Small RNA-sequencing of miRNA present in plasma samples from three individuals.

**A)** Workflow for sample preparation and analysis. **B) & C)** Plasma samples from two individuals were set at room temperature for either 0 or 24 hours, and miRNAs from the plasma were analyzed.

**B)** Principal component analysis (left) and volcano plot (right) comparison of sample A at 0 hours to sample A at 24 hours. **C)** Comparison of sample B at 0 hours to sample B at 24 hours.

**D)** Blood was drawn and set at room temperature for either 0 or 2 hours, and then plasma was extracted and miRNAs from the plasma were analyzed. Comparison of sample C at 0 hours to sample C at 2 hours. Differential expression analysis of miRNA transcriptome is summarized as a volcano plot with an FDR rate of <0.05 and fold change of ≥2. Down-regulated miRNAs are depicted in blue and up-regulated miRNAs are depicted in red.

**Figure S4**. miRNA Ranking by Expression Levels in Counts per Millions (CPM).

**A)** Out of 2,500 miRNAs, the proportion of detected and undetected miRNAs.

**B)** Detected miRNAs are ranked based on their baseline expression levels, from lowest to highest in Counts per Million (CPM). The expression levels are categorized into bins (0–2, 2–4, 4–8, and 8–16 CPM) to illustrate the distribution of miRNA abundance.
